# HANDS2: accurate assignment of homoeallelic base-identity in allopolyploids despite missing data

**DOI:** 10.1038/srep29234

**Published:** 2016-07-05

**Authors:** Amina Khan, Eric J. Belfield, Nicholas P. Harberd, Aziz Mithani

**Affiliations:** 1 Department of Biology, Syed Babar Ali School of Science and Engineering, Lahore University of Management Sciences (LUMS), D.H.A., Lahore 54792, Pakistan; 2 Department of Plant Sciences, University of Oxford, Oxford OX1 3RB, United Kingdom

## Abstract

Characterization of homoeallelic base-identity in allopolyploids is difficult since homeologous subgenomes are closely related and becomes further challenging if diploid-progenitor data is missing. We present HANDS2, a next-generation sequencing-based tool that enables highly accurate (>90%) genome-wide discovery of homeolog-specific base-identity in allopolyploids even in the absence of a diploid-progenitor. We applied HANDS2 to the transcriptomes of various cruciferous plants belonging to genus *Brassica*. Our results suggest that the three C genomes in *Brassica* are more similar to each other than the three A genomes, and provide important insights into the relationships between various *Brassica* tetraploids and their diploid-progenitors at a single-base resolution.

Allopolyploidy is an important evolutionary process in plants which involves interspecific hybridization of two or more differentiated genomes as well as genome doubling^1^. As a result, allopolyploid genomes consist of two or more homeologous subgenomes that have high sequence similarity. This makes it difficult to assign individual sequences to the specific subgenome from which they are derived. Nevertheless, despite their extensive sequence relatedness, the subgenomes present in a polyploid genome evolve over time and diverge in sequence from their common ancestor, resulting in positions in a polyploid genome where the homeologous subgenomes have different bases^1^,^2^. This is in addition to the nucleotide differences accumulated by the diploid-progenitors since their own divergence which are carried forward to the polyploid subgenomes at the time of polyploidization^3^,^4^. Collectively, these base differences between the subgenomes within a polyploid genome are termed as Homeolog-Specific Polymorphisms (HSPs; Fig. 1a)^5^. HSPs are the genetic markers of choice in many transcriptomic and evolutionary studies involving polyploids and have been used to characterize homeolog-specific gene-expression^3^,^4^,^6^. However, this has been done for a limited number of genes due to the complexity arising as a result of genome-wide duplication as well as the cost associated with the process^7^.

With the advent of next-generation sequencing (NGS), it is now possible to survey the whole polyploid genome at a single-base resolution for positions where the individual subgenomes differ from each other^8^,^9^. We have previously developed a tool ‘HSP Assignment using NGS data through Diploid Similarity’ (HANDS) that uses the RNA-seq data for the polyploid and its progenitor-diploids, and characterizes HSPs with an accuracy of >90%^5^. Similarity with progenitor-diploids has recently been exploited to classify gene assemblies in bread wheat (*Triticum aestivum*)^8^ and rapeseed (*Brassica napus*)^9^ subgenomes, and led to the development of tools like PolyCat^10^ and PolyDog^11^ for classification of sequencing reads in allopolyploid cotton. Despite its high predictive accuracy, the applicability of HANDS as well as that of other tools is, however, limited by the fact that they require single-base substitution data for all diploid-progenitors for the characterization of homoeallelic base identities. This is a major limitation since in some cases a diploid-progenitor may be unknown or the genome/transcriptome are unsequenced, as in the case of some *Brassica* species (see below). Also, the existing tools support only up to three diploid-progenitors and hence cannot be used to study complex polyploids such as strawberry and sugarcane, which have four to six diploid-progenitors^12^,^13^.

To address these limitations, we have developed HANDS2, a significantly improved tool than its predecessor that characterizes homoeallelic base-identities with high accuracy even in the absence of RNA-seq data for one of the diploid-progenitors. It also supports up to ten diploid-progenitors allowing it to analyze a wide range of natural as well as synthetic allopolyploids. This restriction to ten diploid-progenitors is due to the fact that there are no known allopolyploids with more than ten diploid-progenitors. The underlying architecture of HANDS2 is designed such that it is able to analyse allopolyploids containing any number of homeologous subgenomes provided sufficient computational resources are available. We have used HANDS2 to study the relationship between various cruciferous plants belonging to genus *Brassica* thus providing important insights into the relationship between different *Brassica* tetraploids and their diploid-progenitors.

## Results and Discussion

### The HANDS2 framework

**Figure 1:**
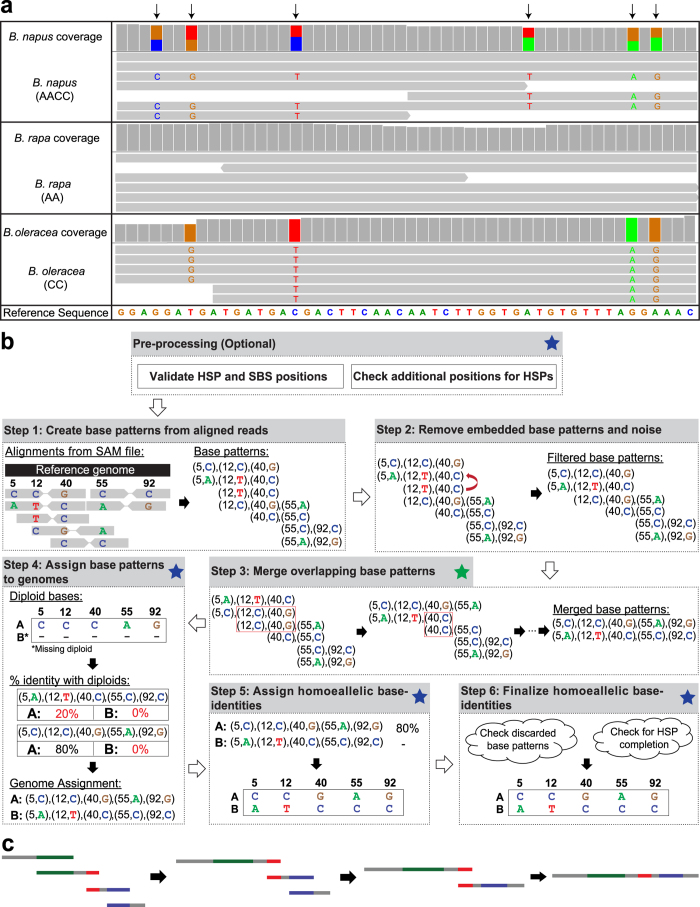
Characterization of homoeallelic base-identities using HANDS2. (**a**) Illustration of Homeolog-Specific Polymorphisms (HSPs) in the *B. napus* genome using RNA-seq data. RNA-seq reads from *B. napus* (allotetraploid) and the two progenitor-diploids (*B. rapa* and *B. oleracea*) were aligned against the *B. rapa* transcriptomic reference sequence. Bases that match the reference sequence are shown in grey and base substitutions (versus the reference sequence) are shown in other colours. HSP positions are marked with arrows. (**b**) An example of using HANDS2 to assign homoeallelic base-identities at HSP positions. HANDS2 takes sequencing alignment/mapping (SAM) file of the polyploid, start and end coordinates of genes/contigs, list of HSPs in the polyploid in conjunction with the lists of single base substitutions (SBSs) in the diploid-progenitors and optional coverage files (for the validation of HSPs and SBSs) as input to assign homoeallelic base-identities in the polyploid using a six-step algorithm: creation of base patterns from aligned reads; filtering of potential sequencing errors and embedded base patterns; iterative merging of overlapping base patterns; assignment of base patterns to subgenomes; assignment of bases to subgenomes using the assigned base patterns; and finalization of base assignments to subgenomes. A green star indicates a new step introduced in HANDS2 whereas a blue start indicates an improvement in HANDS2 over HANDS (see text for details). (**c**) Iterative merging of overlapping base patterns. The base patterns are depicted as grey lines with colours indicating the overlap between different patterns. The patterns are iteratively merged such that the two patterns with the longest overlap are merged first followed by the second longest overlap and so on.

HANDS2 involves comparative alignments of next-generation sequencing (NGS) reads from polyploid and diploid-progenitors onto a suitable reference sequence and uses diploid similarity to assign base-identities to subgenomes at HSP positions. It is able to characterize homoeallelic base-identities with high accuracy even in the absence of RNA-seq data for one of the diploid-progenitors and supports up to ten diploid-progenitors (see above). For this, HANDS2 first creates base patterns (sequence of the pairs (position, nucleotide) for all HSPs found in a read/read-pair) from the NGS reads and then assigns these base patterns, and subsequently subgenome-specific base-identities, to individual subgenomes based on their similarity with the diploids (Fig. 1b). To achieve high accuracy when dealing with missing data, HANDS2 provides a unique option of iteratively merging the overlapping base patterns, an option not available in HANDS, to obtain longer base patterns that have a higher chance of unambiguous assignment to one of the subgenomes than the original base patterns (see below).

#### HANDS2 Input

HANDS2 uses the position-sorted sequence alignment/mapping (SAM)^14^ file of the polyploid genome obtained using sequence alignment tools such as BWA^15^, BOWTIE^16^ or BOWTIE2^17^ along with VCF files containing lists of HSPs and single base substitutions (SBSs) present in the polyploid and the diploid-progenitors respectively to assign base-identity to the polyploid subgenomes. The VCF files can be obtained using standard variant calling tools such as SAMtools^14^, GATK^18^ or FreeBayes^19^. HANDS2 uses VCF version 4.0 or greater unlike HANDS, which used a non-standard format for HSP and SBS lists. HANDS2 also requires a General Feature Format (GFF) file (http://www.sequenceontology.org/gff3.shtml) containing start and end coordinates for each gene/contig. This file is automatically generated by HANDS2 when a transcriptomic reference is constructed from a set of unigenes/contigs using ‘seq2ref’ command. HANDS2 only uses entries of the ‘gene’ type from the GFF file when assigning homoeallelic base-identities.

HANDS2 also accepts optional base coverage files containing number of reads supporting a particular base at each position in a tab-delimited format for HSP/SBS validation and an optional list of positions in the reference (a tab delimited file containing the sequence/chromosome names, positions and reference base) to be checked for HSPs in addition to the positions present in the HSP list during pre-processing step. Base coverage files can be generated from a SAM file using ‘coverage’ command available in HANDS2.

#### Data Pre-processing

HSP characterization can be preceded by an optional pre-processing step, which validates the lists of HSPs and SBSs provided as input. The pre-processing step can be instigated by providing the base coverage files (see above) containing the number of reads supporting a particular base at each reference position as a part of the input for one or more genomes. A base must be present in at least 5% of the reads present at an HSP position for it to be considered as valid subgenome base whereas at least 30% of the reads must support a diploid base for it to be considered as a part of an SBS. Both these cut-offs are user-driven parameters in HANDS2 (unlike HANDS) thus providing a better control to the users. In addition to the supplied HSP positions, HANDS2 also checks for the HSPs at positions containing SBSs in one or more diploid-progenitors, unlike its predecessor where diploid positions were not checked for HSPs, resulting in a higher number of characterized positions than before (Table 1). HANDS2 also accepts an optional list of positions as input to be checked for HSPs in addition to the positions present in the HSP list as a part of pre-processing. This option is not available in HANDS and allows the user to study and compare HSPs across multiple polyploids as in the case of *Brassica* species (see below).

#### Base characterization using HANDS2

HANDS2 characterizes homoeallelic base-identities using a six-step algorithm (Fig. 1b). These are described below.

*Step 1*: Creation of Base Patterns

First, base patterns are created from the aligned reads. A base pattern is a sequence of pairs (position, nucleotide) for all HSPs found in a read-pair (or a read for single-end sequencing). Duplicate patterns are removed and a count of reads is kept for each unique base pattern.

*Step 2*: Filtering of base patterns and removal of embedded patterns

In the second step, base patterns containing a base pair (bases present at two consecutive HSP positions) that is present in less than 5% (a user-specified parameter) of the reads are filtered to remove noise from the data. Furthermore, base patterns that are embedded within another base pattern are also removed.

*Step 3*: Iterative merging of overlapping base patterns

This is a new feature introduced in HANDS2. After removing the noise from the data, the overlapping base patterns are iteratively merged (Fig. 1c). This is done as follows. The base patterns are first sorted in descending order on size and then the pairwise overlap is calculated between them. The two base patterns with the longest overlap are merged together and the resulting base pattern is added to the list of base patterns replacing the original patterns. The base patterns are resorted on size and the process is continued until no more base patterns can be merged. This is the default mode of HANDS2 when dealing with missing diploid and allows it to achieve high accuracy but can be turned off by the user, if desired.

*Step 4*: Assignment of base patterns to subgenomes

In the fourth step, the merged base patterns are assigned to subgenomes based on their similarity to the diploid bases. A base pattern is assigned to a subgenome if at least 50% (a user-specified parameter) bases match with the corresponding diploid genome. When a diploid-progenitor is missing, HANDS2 exploits the fact that a base pattern must come from one of the subgenomes of the polyploid and consequently assigns the base pattern that is not assigned to any subgenome (due to low diploid-identity) to the subgenome corresponding to the missing diploid-progenitor.

*Step 5*: Assignment of base-identities to subgenomes

Once the base patterns are assigned to each subgenome, HANDS2 checks each position in turn and assigns bases to the subgenomes using the already assigned base patterns. In the case of more than one base being present at an HSP position, the base belonging to the base pattern having the maximum percentage identity with the diploid is assigned at that position. HANDS2 introduces a new option of using additive mode instead of the default maximum mode for base assignment whereby a base having the highest sum of percentage identities across all base patterns containing the base is assigned at that HSP position. In both modes, no base assignment is made at a position in the case of a tie. Once all positions have been processed, all base patterns are rechecked for consistency and those that conflict with the assigned bases are removed. This step is repeated until no more bases can be assigned.

*Step 6*: Finalization of base-identities

In the sixth and the final step, the base assignments to the subgenomes are finalized using the inherent information from HSPs and NGS platform. Since all the bases present at an HSP position must belong to one of the polyploid subgenomes, a base that is left unassigned at a position is allocated to a subgenome if all the remaining bases have been assigned to other subgenomes. This additional step, which is not present in HANDS, results in a higher number of fully characterized positions by HANDS2 compared to HANDS. This step, however, may lead to incorrect base assignments at positions where the polyploid genome contains allelic polymorphism within a subgenome and can be turned off by the user when dealing with heterozygous species. In this step, base patterns that are left unassigned or were removed in the previous step are also rechecked for base assignment by calculating their percentage identity with the already assigned bases.

#### HANDS2 output

HANDS2 writes the base assignments for all subgenomes as standard VCF files and reports all positions where one or more subgenome has been assigned a base-identity. This in the default output format in HANDS2 unlike HANDS, which only allows tab-delimited format. VCF output enables a user to use the HANDS2 output as an input to other tools. For example, ‘FastaAlternateReferenceMaker’ command in GATK^18^ could be used to create homeolog-specific fasta file using the VCF files. HANDS2 also provides an option to generate the tab-delimited output with each file containing the name of reference sequence, HSP position, reference base, diploid base (‘0’ for no coverage, ‘<’ for low coverage, ‘*’ for ambiguous/heterozygous base and ‘?’ for missing diploid), assigned base in the subgenome and a yes/no flag to indicate fully characterized positions (feature not available in HANDS) for all positions where one or more subgenome has been assigned a base-identity. The tab-delimited output allows a direct comparison between polyploid subgenomes and their diploid progenitors.

### Assessment of HANDS2 performance

To evaluate the performance of HANDS2, we analyzed the high-throughput RNA sequencing (RNA-seq) data for hexaploid bread wheat and its diploid-progenitors generated for the validation of HANDS^5^, and compared the results, with and without enabling the support for missing genome in HANDS2, to those obtained using HANDS. HANDS2 was able to characterize 20% more HSP positions than HANDS with similar accuracy (>96%) for wheat chromosomes 1 and 5 (Table 1 and Methods). Even when *Aegilops speltoides*, the distant donor of B-subgenome in bread wheat, was specified as missing genome, we obtained the same accuracy level (>96%) (Table 1) thus demonstrating highly predictive accuracy of HANDS2 even in the absence of complete data.

### Characterization of base-identities in *Brassica* tetraploids

**Figure 2:**
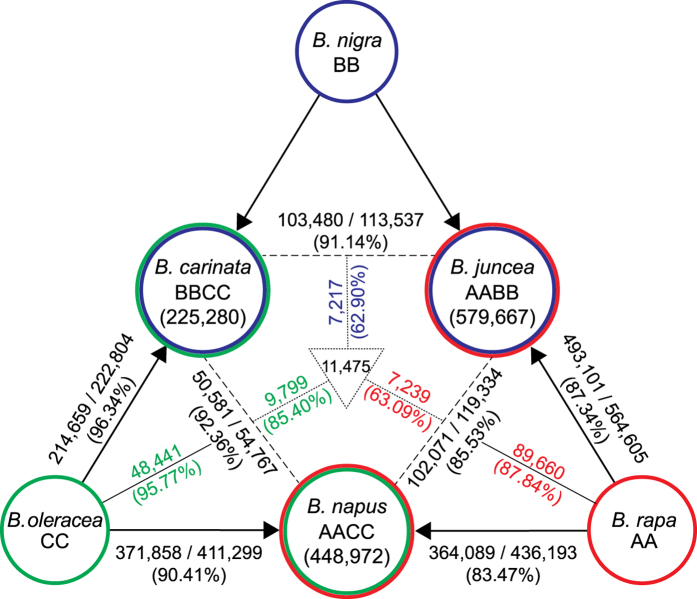
Characterization of homoeallelic base-identities in *Brassica*. Diploid species are depicted using single circles whereas tetraploids are shown with double circles with colours corresponding to their diploid-progenitors. The arrows represent the relationships between different tetraploid subgenomes and their corresponding diploid-progenitors whereas the dashed lines represent the relationships between corresponding subgenomes in different tetraploids. The first number along these lines represents the number of shared bases between a subgenomes and its diploid-progenitor (arrow), and between the two subgenomes (dashed line) whereas the second number represents the number of shared positions between them. The number inside the dotted triangle is the number of HSP positions shared between all three tetraploids and the numbers in colours along the dotted lines represent the number of shared bases in the corresponding subgenomes (A: red, B: blue, and C: green) at these common positions. The coloured numbers along the solid lines represent the number shared bases between the two subgenomes and the corresponding diploid-progenitor.

We next used HANDS2 to characterize different cruciferous plants belonging to genus *Brassica*. In *Brassica*, three diploids species (*Brassica rapa*, AA; *Brassica nigra*, BB; and *Brassica oleracea*, CC) have paired up in all possible combinations giving rise to three tetraploid species (*Brassica napus*, AACC; *Brassica juncea*, AABB; and *Brassica carinata*, BBCC), known as ‘Triangle of U’^20^,^21^ (Fig. 2). The unavailability of RNA-seq data for *B. nigra* has so far prevented the study of relationships between the three tetraploids at a single-base resolution since base assignments cannot be made in *B. juncea* and *B. carinata*, which have *B. nigra* as B diploid-progenitor. We applied HANDS2 on published transcriptomic sequencing datasets of different *Brassica* species (Supplementary Table S1), which were aligned against an *in silico B. rapa* transcriptomic reference constructed using Ensembl Plants build 1.27 (http://plants.ensembl.com) (Supplementary Fig. S1 and Methods). We first characterized base-identities in *B. napus* since data for both its diploid-progenitors is available. HANDS2 reported a total of 495,164 HSP positions out of which 448,972 (91.8%) positions were fully characterized, i.e. base-identities were assigned to both subgenomes (Fig. 2, Table 2 and Supplementary Table S2). When either *B. rapa* or *B. oleracea* was designated as missing genome, 467,321 (94.4%) and 461,528 (93.2%) positions respectively were fully characterized. Out of these fully characterized positions, ~92% positions had the same homoeallelic base assignments as those obtained when both the diploid-progenitors were specified. HANDS, on the other hand, reported only 401,653 HSP positions in *B. napus* out of which 319,239 (79.5%) positions were fully characterized. Out of these, 294,279 (92.1%) positions were present in the list of fully characterized positions reported by HANDS2 with ~94% positions having the same base-identities assigned to the two subgenomes by both the tools. To test whether the use of *B. rapa* as the reference sequence had resulted in any bias, we repeated the above analysis using *B. oleracea* as the transcriptomic reference sequence (Table 2). No significant difference was found (G-test, P-value≈1) suggesting that base assignments made by HANDS2 were independent of the choice of the reference sequence.

We subsequently characterized homoeallelic base-identities in the remaining two tetraploids *B. juncea* and *B. carinata* resulting in a total of 579,667 and 225,280 fully characterized HSP positions, respectively (Fig. 2, Supplementary Tables S3 and S4). Given the high accuracy level of HANDS2 on *T. aestivum* and *B. napus* genomes, it is safe to assume that these base assignments are of high quality even in the absence of *B. nigra* RNA-seq data. The low number of HSP characterization in *B. carinata* is likely due to the low coverage of sequencing reads resulting in a number of HSP positions being ignored (see Methods) as well as unavailability of paired-end sequencing data (Supplementary Table S1), which results in shorter base patterns having a lower chance of unambiguous assignment to subgenomes compared to the paired-end data.

### Analysis of relationship between different *Brassica* tetraploids and their diploid-progenitors

Once the homoeallelic base-identities were characterized, we analyzed the relationship between different *Brassica* tetraploids and their diploid-progenitors. *B. napus* subgenomes were found to have lower similarity with their diploid-progenitors (83.5% for A and 90.4% for C subgenomes) compared to *B. juncea* (87.3% for A subgenome) and *B. carinata* (96.3% for C subgenome) (Fig. 2, Supplementary Tables S5–S8). Also, the number of shared bases between the *B. napus* A subgenome and *B. rapa* were significantly lower than the bases shared between the *B. napus* C subgenome and *B. oleracea* (G-test, P-value < 1.6 × 10^−16^). Interestingly, a higher gene loss in the A subgenome compared to C subgenome has been reported in *B. napus* recently^9^. The highest level of similarity between *B. carinata* C subgenome and *B. oleracea* can be attributed to the recent origin^22^ and low genetic diversity of the tetraploid^23^. Next we evaluated the positions that were shared between different tetraploids (Fig. 2 and Supplementary Tables S9–S11). The two C subgenomes had the highest similarity to each other with 50,581 out of 54,767 shared HSP positions (92.4%) having the same base. Out of these, 95.8% positions were also shared with *B. oleracea* (Supplementary Table S11). However, the A subgenomes were least similar to each other with 102,071 out of 119,334 positions (85.5%) having the same base and shared 87.8% positions with *B. rapa* (Supplementary Table S9). To test if the use of both diploid-progenitors for *B. napus* versus single diploid-progenitors for the other two tetraploids while assigning homoeallelic base-identities had resulted in any bias, we repeated the whole analysis using a single diploid-progenitor for *B. napus* (*B. oleracea* and *B. rapa* designated as missing when comparing A and C subgenomes respectively). The two C subgenomes were still found to be more similar to each other with 54,100 out of 56,634 HSP positions (92.3%) having the same base compared to the two A subgenomes where only 106,819 out of 126,680 positions (84.3%) had the same base. Finally, we studied the positions that were shared between all three tetraploids (Fig. 2 and Supplementary Table S12). Again, the two *Brassica* C subgenomes had the highest similarity amongst each other suggesting a significantly higher conservation in C subgenomes compared to A and B subgenomes (G-test, P-value < 1.6 × 10^−16^). Collectively, these observations suggest that *Brassica* C genomes are more similar to each other than the A genomes.

## Conclusion

In summary, HANDS2 provides a highly accurate approach to genome-wide discovery of homoeallelic base identities in allopolyploids and works even in the absence of a diploid-progenitor. HANDS2 is implemented in Java and is available for download at https://genomics.lums.edu.pk/software/hands2/ with a user manual and test datasets from *T. aestivum* and *B. napus* genomes. Since HANDS2 uses read alignments to characterize HSP bases, it requires the RNA-seq data to be of high quality with sufficient coverage to be able to accurately identify and assign the base-identities. Also, like other current tools that work on diploid similarity^5^,^10^,^11^, it has difficulty in detecting instances where a gene is silenced in one or more subgenomes, and may therefore incorrectly assign base-identities at silenced positions. Similarly, cases like gene conversion or homeologous exchanges, which are frequent in polyploids^9^,^24^, may also result in biased base assignments. Nevertheless, the ability to accurately assign base-identities at non-silenced positions despite missing data and the support for up to ten diploid-progenitors make HANDS2 a viable approach for HSP characterization in polyploids thus enabling important insights into the complex genome architecture and evolution of polyploids at a single-base resolution.

## Methods

### Assessment of HANDS2 accuracy

We tested the accuracy of base assignments made by HANDS2 using the high-throughput RNA sequencing (RNA-seq) data for hexaploid bread wheat (*T. aestivum*; AABBDD) and its diploid-progenitors (*Triticum urartu*; AA, *Aegilops speltoides*; BB, and *Aegilops tauschii*; DD), which was generated for the validation of HANDS^5^. The sequencing data for the polyploid and diploid-progenitors was first aligned, filtered and variants were called (see below). Base characterization was subsequently done using HANDS and HANDS2. To test the accuracy of the tool, especially in the case of missing genome, we used the RNA-seq data for wheat chromosomes 1 and 5 nullisomic-tetrasomic (NT) lines^5^,^26^. Wheat NT lines are a set of lines each missing a single chromosome (nullisomic) which is substituted by an additional copy of a homeologous chromosome (tetrasomic)^25^, and provide an ideal framework, albeit at a very high cost, to accurately characterize wheat HSPs at the genome-wide level^5^,^26^. The base assignments made by HANDS and HANDS2 were evaluated against those obtained using these NT lines^5^.

### Construction of *B. rapa* and *B. oleracea in silico* transcriptomic references

The *B. rapa* and *B. oleracea* transcriptomic references were constructed using Ensembl Plants build 1.27 (http://plants.ensembl.com) containing 41,393 and 59,225 cDNA sequences respectively. The *in silico* transcriptomic references were obtained using ‘seq2ref’ command in HANDS2, which concatenates the contigs/cDNA sequences such that two consecutive sequences are separated by a gap of 200 (a user specified parameter) Ns (Supplementary Fig. S1). The *B. rapa* reference comprised of a total of 56,434,134 bases out of which 8,278,800 bases were ‘N’s used as separators. Similarly, *B. oleracea* reference contained a total of 73,571,942 bases with 11,845,000 ‘N’s.

### Sequence alignment, filtering and visualization

Whole transcriptome sequencing (RNA-seq) reads for *T. aestivum* were mapped to the *T. aestivum* Unigene build 60 reference^5^ whereas the reads for *Brassica* species (Supplementary Table S1) were mapped to *B. rapa* and *B. oleracea* transcriptome 1.27 references using Burrows-Wheeler Aligner (BWA)^15^ using default parameters (Supplementary Tables S13 and S14). Reads with low mapping quality (phred score ≤20) were filtered out using custom scripts written in C++. Additionally, for paired-end data, reads for which the only one read in a pair was aligned as well as those reads which mapped in a different gene than their mates were also removed. The alignments were visualized using Integrative Genome Viewer (IGV; Fig. 1a)^27^.

### Variant calling and filtering

The filtered alignments were used to generate pileups using SAMtools^14^ version 0.1.19 ‘mpileup’ command with probabilistic realignment for the computation of base alignment quality (BAQ) disabled, and the minimum and maximum coverage thresholds set to 3 and 50,000 respectively. These pileup files were then used to call variants (HSPs for the polyploid and single base substitutions (SBSs) for the progenitor-diploids) using bcftools version 0.1.19 ‘view’ command (available as part of SAMtools) using default parameters. The variant lists were subsequently filtered using ‘varFilter’ command of vcfutils.pl with parameters ‘-1 0 -4 0 -d 3 -D 50000’ available in SAMtools version 0.1.19 to remove potential false positives including errors arising during DNA sequencing itself. Variants with low quality (phred score ≤20) were also removed. For diploids, ambiguous (heterozygous) base calls were also ignored.

## Additional Information

**How to cite this article**: Khan, A. *et al*. HANDS2: accurate assignment of homoeallelic base-identity in allopolyploids despite missing data. *Sci. Rep.* **6**, 29234; doi: 10.1038/srep29234 (2016).

## Supplementary Material

Supplementary Information

Supplementary Tables S1,S13-14

Supplementary Table S2

Supplementary Table S3

Supplementary Table S4

Supplementary Table S5

Supplementary Table S6

Supplementary Table S7

Supplementary Table S8

Supplementary Table S9

Supplementary Table S10

Supplementary Table S11

Supplementary Table S12

## Figures and Tables

**Table 1:** Performance comparison of HANDS2 versus HANDS using *T. aestivum* data.

Chromosome	Missing Diploid	Subgenome	HANDS			HANDS2		
			Positions Assigned	Filtered Positions*	Correct Assignments§	Positions Assigned	Filtered Positions*	Correct Assignments§
Chr 1	None	A	28,324	26,478 (93.48%)	26,005 (98.21%)	34,154	32,756 (95.91%)	32,079 (97.93%)
		B	27,453	25,555 (93.09%)	24,714 (96.71%)	32,943	31,456 (95.49%)	30,408 (96.67%)
		D	28,788	26,592 (92.37%)	26,287 (98.85%)	34,727	33,220 (95.66%)	32,690 (98.40%)
	*Ae. speltoides* (BB)	A				32,989	32,005 (97.02%)	31,237 (97.60%)
		B	Option not available			33,492	29,273 (87.40%)	28,171 (96.24%)
		D				33,439	32,499 (97.19%)	31,958 (98.34%)
Chr 5	None	A	34,553	32,166 (93.09%)	31,677 (98.48%)	42,209	40,339 (95.57%)	39,539 (98.02%)
		B	33,499	31,140 (92.96%)	30,141 (96.79%)	40,825	38,970 (95.46%)	37,560 (96.38%)
		D	36,363	33,435 (91.95%)	33,053 (98.86%)	44,406	42,545 (95.81%)	41,823 (98.30%)
	*Ae. speltoides* (BB)	A				40,904	39,522 (96.62%)	38,762 (98.08%)
		B	Option not available			42,802	37,262 (87.06%)	35,913 (96.38%)
		D				43,043	41,714 (96.91%)	41,031 (98.36%)

^*^Positions where all HSP bases were assigned to the three sub-genomes, the genome was not silenced in the hexaploid and the diploid had an unambiguous base with read coverage ≥3.

^§^Assignments were evaluated against the assignments made using nullisomic-tetrasomic lines. See text for details.

**Table 2:** HSP characterization in *B. napus* using HANDS2.

Reference	Missing Diploid	Subgenome	Positions Assigned	Filtered Positions*	Correct Assignments§
*B. rapa* 1.27	None	A/C	495,164	448,972 (90.67%)	–
	*B. oleracea*	A/C	495,164	467,321 (94.38%)	430,030 (92.02%)
	*B. rapa*	A/C	495,164	461,528 (93.21%)	424,215 (91.92%)
*B. oleracea 1.27*	None	A/C	502,716	458,244 (91.15%)	–
	*B. oleracea*	A/C	502,716	467,382 (92.97%)	432,353 (92.51%)
	*B. rapa*	A/C	502,716	474,560 (94.40%)	439,153 (92.54%)

^*^Positions where all HSP bases were assigned to the two sub-genomes. These positions were used for further analysis.

^§^Compared to complete dataset.
