# Supplementary material for: HANDS2: accurate assignment of homoeallelic base-identity in allopolyploids despite missing data
Source: Sci Rep. 2016 Jul 5;6:29234. doi: 10.1038/srep29234 (PMC4932600; doi:10.1038/srep29234)
Supplement: Supplementary Information [file srep29234-s1.doc]

**SUPPLEMENTARY INFORMATION**

**HANDS2: accurate assignment of homoeallelic base-identity in allopolyploids despite missing data**

Amina Khan^1^, Eric J Belfield^2^, Nicholas P Harberd^2^, Aziz Mithani^1,*^

^1^Department of Biology, Syed Babar Ali School of Science and Engineering, Lahore University of Management Sciences (LUMS), D.H.A., Lahore 54792, Pakistan;

^2^Department of Plant Sciences, University of Oxford, Oxford OX1 3RB, United Kingdom.

*Corresponding author: [aziz.mithani@lums.edu.pk](mailto:aziz.mithani@lums.edu.pk)

**Figure S1. Creation of *in silico* *B. rapa* transcriptomic reference.** The *B. rapa* transcriptomic reference was constructed using Ensembl Plants build 1.27 ([http://plants.ensembl.com](http://plants.ensembl.com/)) containing 41,393 cDNA sequences. The reference was constructed by concatenating the sequences such that two consecutive sequences were separated by a gap of 200 N’s. The reference was a total of 56,434,134 bases in length out of which 8,278,800 bases were ‘N’s used as separators.
